# Feasibility of measuring fusional vergence amplitudes objectively

**DOI:** 10.1371/journal.pone.0284552

**Published:** 2023-05-04

**Authors:** Cristina Rovira-Gay, Clara Mestre, Marc Argiles, Valldeflors Vinuela-Navarro, Jaume Pujol

**Affiliations:** 1 Centre for Sensors, Instruments, and Systems Development (CD6), Universitat Politècnica de Catalunya (UPC), Terrassa, Spain; 2 School of Optometry, Indiana University, Bloomington, IN, United States of America; Cairo University Kasr Alainy Faculty of Medicine, EGYPT

## Abstract

Two tests to measure fusional vergence amplitudes objectively were developed and validated against the two conventional clinical tests. Forty-nine adults participated in the study. Participants’ negative (BI, base in) and positive (BO, base out) fusional vergence amplitudes at near were measured objectively in an haploscopic set-up by recording eye movements with an EyeLink 1000 Plus (SR Research). Stimulus disparity changed in steps or smoothly mimicking a prim bar and a Risley prism, respectively. Break and recovery points were determined offline using a custom Matlab algorithm for the analysis of eye movements. Fusional vergence amplitudes were also measured with two clinical tests using a Risley prism and a prism bar. A better agreement between tests was found for the measurement of BI than for BO fusional vergence amplitudes. The means ± SD of the differences between the BI break and recovery points measured with the two objective tests were -1.74 ± 3.35 PD and -1.97 ± 2.60 PD, respectively, which were comparable to those obtained for the subjective tests. For the BO break and recovery points, although the means of the differences between the two objective tests were small, high variability between subjects was found (0.31 ± 6.44 PD and -2.84 ± 7.01 PD, respectively). This study showed the feasibility to measure fusional vergence amplitudes objectively and overcome limitations of the conventional subjective tests. However, these tests cannot be used interchangeably due to their poor agreement.

## Introduction

Vergence eye movements are disjunctive movements used to align the fovea of each eye with targets located at different distances during binocular viewing [1–3]. These movements are typically described as consisting of four components: tonic, proximal, accommodative, and fusional vergence [4]. Fusional vergence, driven by retinal disparity, is the only component that provides feedback to the vergence system about response accuracy, and its function is to reduce retinal disparity within the limits of Panum’s fusional area to establish accurate binocular fixation of the target of interest [5]. The evaluation of fusional vergence is fundamental in standard optometric exams, as it provides information about the patient’s binocular vision status and, together with the results of other optometric tests, is useful to diagnose binocular vision dysfunctions [6, 7]. The prevalence of non-strabismic binocular vision dysfunctions has been well documented in the literature, although results differ between studies reporting prevalence ranges from 13.5% to 32%; [8–11] 2.29% to 15.81% of these associated with accommodation disorders [8, 10, 11], and 8% to 12.9% related to vergence disorders [8, 12]. Convergence insufficiency (CI) and accommodative insufficiency (AI) are the most common non-strabismic binocular dysfunctions with a prevalence ranging from 3.43% to 10.3% for CI [8, 9, 11, 13] and 11.5% to 5.3% for AI [9, 11].

In clinics, fusional vergence amplitudes are commonly measured with the smooth vergence test using the rotatory Risley prisms mounted in the phoropter, or with the step vergence test using a prism bar. Base-in (BI) prisms are used to measure divergence or negative fusional vergence amplitudes, and base-out (BO) prisms are used to measure convergence or positive fusional vergence amplitudes. To perform these tests, vergence demand is increased until fusion is disrupted (the “break point”). Then, the vergence demand is reduced until fusion is recovered and the patient reports single vision. The prism power at that time is the recovery point [1, 6, 14]. Although these subjective smooth and step tests have a similar procedure, they cannot be used interchangeably due to their low level of agreement [14–17], and their repeatability is poor [16]. The fact that these tests are subjective, because the results depend on patients’ responses and/or on the examiner’s criterion, which can be the case of the step subjective test if the examiner predicts loss of fusion by observing the misalignment of the eyes, could explain these limitations and lead to low intraexaminer and interexaminer reliability [18], high variability of the results, and disagreement about the expected normal values of fusional vergence amplitudes [15, 18–24]. The subjectivity and variability of the results depending on the patient’s and examiner’s criteria could be overcome by measuring fusional vergence amplitudes objectively with eye tracking systems. Moreover, objective recordings of vergence eye movements would allow a more detailed characterization of the physiology of eye movements compared with traditional optometric clinical testing [25, 26].

Fusional vergence amplitudes have been measured objectively in previous studies. Some studies have used objective measures to determine changes in fusional vergence amplitudes after different vision therapy protocols [25, 27, 28]. Sreenivasan et al. (2016) measured fusional vergence break points objectively in a subgroup of adult participants using a Purkinje image eye tracker and eccentric photorefractor (the PowerRefractor, MultiChannel Systems) and a prism bar, and compared the results with simultaneous subjective reports of diplopia. They concluded that both objective measures and subjective reports were in good agreement [29]. In a recent study, Gao et al. (2022) measured fusional vergence objectively using a Tobii 4C eye tracker and presenting stimuli on a monitor while each participant wore 3D frame-sequential stereo glasses. They also found good agreement between objective and subjective simultaneous measures of break and recovery points, with an offset reflecting participants’ reaction times [30].

In order to overcome the actual limitation of subjectivity of the methods used in clinics, the goal of this cross-sectional study was to implement two different objective tests to measure fusional vergence amplitudes at near where vergence demand increased in small steps, mimicking the step vergence test, and smoothly, mimicking the smooth vergence test, in an haploscopic set-up. Fusional vergence amplitudes were also measured subjectively using a prism bar and a rotatory Risley prism and the agreement between tests was examined. To our knowledge, this is the first study to compare fusional vergence amplitudes measured objectively and subjectively with the smooth and step tests.

## Methods

### Participants

A total of 49 young adults (mean age ± standard deviation (SD): 23.22 ± 3.06 years) (32.7% male, 67.3% female), mainly university students, were recruited from the local area to participate in this cross-sectional study. Most of them were not familiar with fusional vergence testing. The inclusion criteria were participants between 19 and 29 years old, spherical refractive errors between + 5.00 D and—5.00 D, astigmatism lower than 2.00 D, anisometropia lower than 1.25 D, corrected visual acuity of 0.05 LogMAR or better in the two eyes at distance and near, no history of ocular pathology or ocular surgery, absence of strabismus determined with the cover test, and no use of active orthokeratology treatment. Both spectacle and contact lens wearers were included in the study. All participants wore their habitual refractive correction during the experiment. All participants received a standard binocular vision examination, including an alternate cover test with prism neutralization at near, near point of convergence, binocular accommodative facility with +2.00/-2.00 D flipper lenses, vergence facility at near with 3 Prism Dioptre (PD) BI and 12 PD BO prisms, and Convergence Insufficiency Symptom Survey (CISS) questionnaire [31].

All participants were informed about the nature of the study and signed a written informed consent form prior to the start of the study. The study followed the tenets of the Declaration of Helsinki and was approved by the Ethics Committee of Hospital Mutua de Terrassa (Terrassa, Spain).

### Equipment and procedure

Positive and negative fusional vergence amplitudes were measured at near (40 cm) with two subjective tests typically used in clinics, the smooth and step subjective tests; and two objective tests, the smooth and step objective tests. The order of the tests was the same for each participant: step and smooth subjective tests, and step and smooth objective tests. To prevent the impact of vergence adaptation, for each test, negative fusional vergence amplitude was measured before positive fusional vergence amplitude [32, 33]. The fixation target used for all tests was a column of 0.20 LogMAR letters in a white background presented at a viewing distance of 40 cm. Participants were asked to look at the stimulus and try to keep it single and clear. For the subjective tests, the target was printed on a cardboard and measures were done with habitual room illumination, whereas for the objective tests, the target was presented on two monitors in an otherwise dark room.

Fusional vergence amplitudes were measured with the step subjective test using a prism bar with prism powers from 1 PD to 40 PD in steps of 2 PD from 2 PD to 20 PD and steps of 5 PD from 25 PD to 40 PD. The prism bar was placed in front of the participant’s right eye, and the prism power was changed every 2 seconds approximately. The rotatory Risley prisms were used for the smooth subjective test. The prisms mounted in the phoropter ranged from 0 to 40 PD and the amount of prism power was changed at an approximate velocity of 1 PD/s. In order to unify the procedure of the two subjective tests, the break and recovery points were recorded as the amount of prism when participants verbally reported diplopia and single vision while vergence demand was increased and decreased, respectively. The same procedure was done with both BI and BO prisms to measure negative and positive fusional vergence amplitudes, respectively. Only one repetition of each subjective test was done.

**Fig 1:**
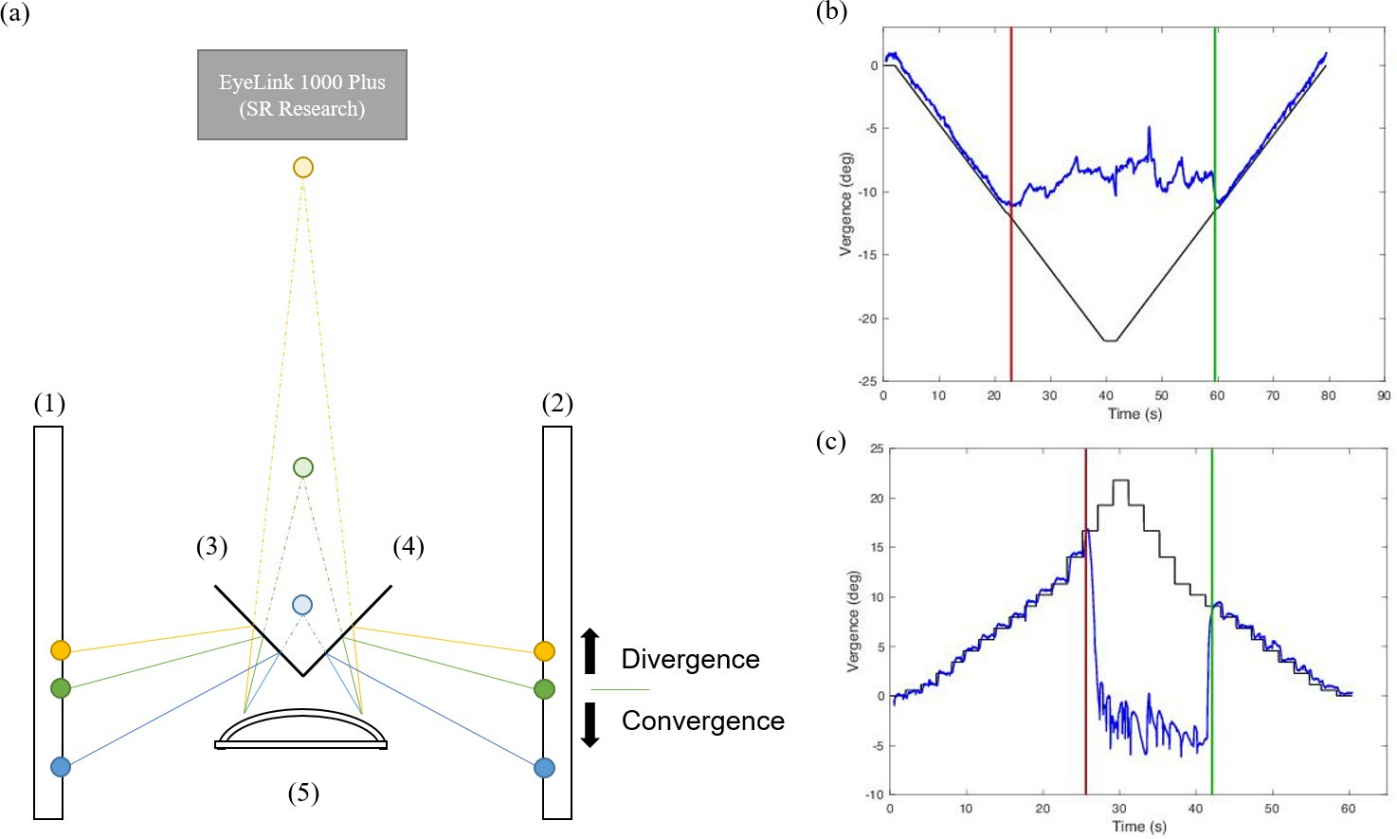
Haploscopic system and representative examples of vergence movements measured with the experimental set-up. (a) Schematic representation of the haploscopic system used in the present study: (1) and (2) monitors, (3) and (4) cold mirrors, and (5) chin rest. The three circles in each screen represent three different stimulus positions. The two green stimuli drive the same convergence angle as a real stimulus placed at 40 cm in front of the subject, represented by the light green circle. Thus, this is the stimuli’s starting position (0 PD). Relative to this position, the yellow and blue stimuli in the two monitors drive fusional divergence and convergence, respectively. (b) and (c) Representative examples of vergence movements measured with EyeLink (in blue) during a negative fusional vergence amplitude measure with the smooth objective test (b), and a positive fusional vergence amplitude measure with the step objective test (c). The vergence demand driven by the stimuli is shown in black. The red and green vertical lines represent the time of the break and recovery points determined objectively.

An haploscopic system was used to measure fusional vergence amplitudes with the step and smooth objective tests (Fig 1A). The set-up consisted of two identical monitors with a resolution of 1920 x1080 pixels (angular pixel size of 2.37 arc min at 40 cm) and frame rate of 60 Hz. Each eye could see the stimulus presented in one of the monitors thanks to two cold mirrors oriented at an angle of 45 deg relative to each monitor. The eye-tracker EyeLink 1000 Plus (SR Research Ltd., Ontario, Canada) was used to measure eye movements at a sampling rate of 500 Hz. The eye-tracker was positioned at its normal operating distance (around 50 cm) in front of the subject and it could track eye movements through the cold mirrors, as they transmitted infrared wavelengths. A chin rest was used to minimize participants’ head movements and fix the viewing distance of 40 cm. The stimulus presentation on each monitor was controlled by a custom software coded in Matlab R2020b (The MathWorks, Inc., Natick, MA, USA) using the Psychophysics Toolbox [34–36].

Before the fusional vergence amplitudes objective measurements, a monocular centering procedure was done to determine the position in each screen that corresponded to a baseline convergence at 40 cm (position of the green stimuli in Fig 1A). This position varied across participants as a function of their interpupillary distance. Then, the EyeLink built-in 9-points calibration and validation procedures were done monocularly. The mean ± SD eye tracking recordings’ spatial accuracy averaged across eyes and participants was 0.56 ± 0.23 deg. Then, the fusional vergence amplitudes measurement started. Fusional convergence and divergence movements were driven by changing the stimuli position synchronously in the two screens from the baseline (0 PD) position as illustrated in Fig 1A. In the smooth objective test, the stimuli moved smoothly at 1 PD/s to increase the vergence demand up to 40 PD to mimic the stimulation with the rotatory Risley prisms during the smooth subjective test. In the step objective test, the stimuli moved every 2 seconds in steps to drive the same vergence demands as the prism bar used in the step subjective test.

In the two objective tests, three repetitions were done consecutively, with a short break between negative and positive fusional vergence amplitudes measures to prevent fatigue. The centering, calibration, and validation procedures were repeated before each fusional vergence amplitudes measurement. The break and recovery points were determined offline using a custom algorithm for the analysis of vergence movements.

### Data analysis

Binocular eye position data were processed offline using Matlab to determine the break and recovery points (Fig 1B and 1C). Periods of 200 ms before and after each blink identified by the EyeLink software were removed and filled by linear interpolation. Vergence was computed by subtracting left and right eyes horizontal gaze positions. Fusional vergence break points were determined with an iterative least-squares fitting procedure. For the smooth objective test, a straight line was fitted to the vergence position over time iteratively, adding 0.10 seconds of data in each iteration. For the step objective test, a second-degree polynomial function was fitted instead of a line as it described better the change in vergence demand over time. Then, the break point was determined as the vergence demand at the time of the last fit before the coefficient of determination of the fit started to decrease. The same procedure was done to determine the recovery point. For the smooth objective test, the mean ± SD coefficient of determination (R^2^) that determined the break and recovery points was 0.979 ± 0.026 for negative fusional vergence and 0.982 ± 0.037 for positive fusional vergence. For the step objective test, the mean coefficients of determination were 0.964 ± 0.036 and 0.980 ± 0.019 for negative and positive fusional vergence, respectively. In this case, the coefficient of determination obtained when fitting the vergence demand over time with a second-degree polynomial was 0.990.

A break point of 40 PD was assigned to participants who did not exhibit loss of motor fusion during the objective tests or who did not report diplopia during the subjective tests. No recovery value was recorded in these cases.

### Statistical analysis

IBM SPSS 27.0 for Windows was used for statistical analysis. A significance level (*p*-value) of less than 0.05 was considered significant. All variables were first examined for normality using the Shapiro Wilk test. Parametric tests were used with normally distributed variables, whereas nonparametric tests were used when normality could not be assumed. Paired t-tests or Wilcoxon tests were used to analyse the differences between tests. The Bonferroni correction was applied to control for type I errors when performing multiple pairwise comparisons [37]. In this case, the Bonferroni correction set the significance level at p < 0.008 (0.05/6 = 0.008). Bland-Altman plots were done to describe the agreement between fusional vergence amplitudes tests [38]. Association between the results obtained with the different tests was determined using Pearson or Spearman’s correlation coefficients. G*Power 3.1.9.7 was used to compute effect sizes [39].

## Results

The results of the binocular vision tests performed before the experimental protocol for the 49 participants were: mean ± SD phoria at 40 cm of 1 PD exophoria ± 5 PD measured with the alternate cover test, near point of convergence of 7 ± 3 cm (break point) and 9 ± 3 cm (recovery point), binocular accommodative facility of 9 ± 4 cycles per minute (cpm), vergence facility of 18 ± 6 cpm and CISS questionnaire score of 12 ± 7.

**Fig 2:**
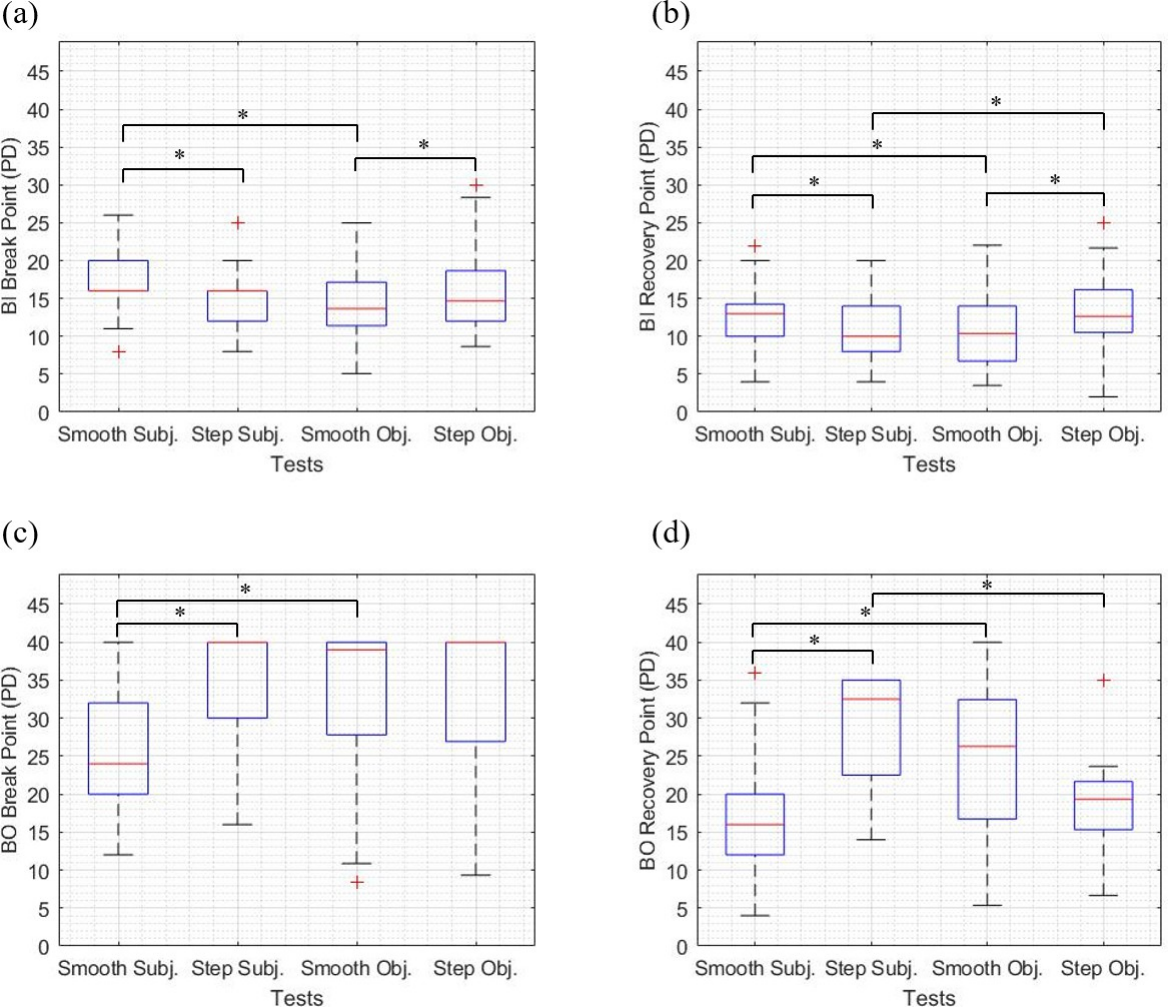
Boxplots of the median and interquartile range (IQR) of the fusional vergence amplitudes measured with the four tests. Base-In (BI) break points (a), BI recovery points (b), Base-Out (BO) break points (c), and BO recovery points (d) measured with the four tests. * indicates statistically significant differences between the tests (corrected *p*-value < 0.008). PD (Prism Dioptre), Subj. (Subjective), and Obj. (Objective).

The median and interquartile range (IQR) of the fusional vergence amplitudes measured with the four tests are shown in Fig 2. For the sake of brevity, negative and positive fusional vergence amplitudes are named after the base of the prism used to measure them, i.e., BI and BO, respectively. The following pairs of tests were compared: (i) subjective tests (smooth and step subjective tests), (ii) objective tests (smooth and step objective tests), (iii) smooth tests (smooth objective and smooth subjective tests), and (iv) step tests (step objective and step subjective tests).

### (i) Smooth and step subjective tests

**Fig 3:**
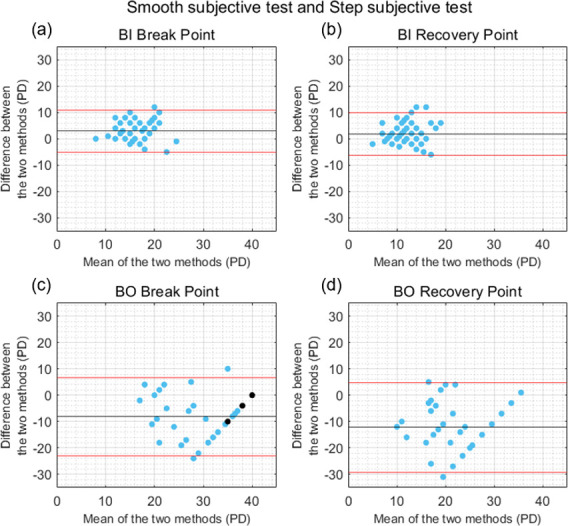
Bland-Altman plots comparing the results obtained with the smooth and step subjective tests. (a) Base-In (BI) break point, (b) BI recovery point, (c) Base-Out (BO) break point, and (d) BO recovery point. Black horizontal lines correspond to the mean of the differences between tests, and red horizontal lines show the 95% limits of agreement. Black dots correspond to the participants who did not exhibit loss of motor fusion and were assigned a break point of 40 PD.

Fusional vergence amplitudes measured with the smooth subjective test were significantly higher than with the step subjective test for the BI break (U = -4.23, p < 0.001, Cohen’s d = 0.72) and recovery (t(48) = 3.08, p = 0.003, Cohen’s d = 0.44) points. The mean ± SD of the differences between the two tests were 2.95 ± 4.05 PD for the BI break point and 1.83 ± 4.17 PD for the BI recovery point (Fig 3A and 3B). The results obtained with the smooth subjective test were significantly lower than with the step subjective test for the BO break (U = -5.18, p < 0.001, Cohen’s d = 1.06) and recovery (U = -5.43, p < 0.001, Cohen’s d = 1.40) points. The mean ± SD of the differences were -8.12 ± 7.60 PD for the BO break point and -12.25 ± 8.69 PD for the BO recovery point (Fig 3C and 3D). Five subjects were excluded from the BO recovery point comparison as they did not report diplopia during the smooth or step subjective tests. Low to moderate significant correlations were found between the smooth and step subjective tests measures of the BI break point (*rho* = 0.37, *p* = 0.009), BI recovery point (*r* = 0.48, *p* = 0.004), BO break point (*rho* = 0.57, *p* < 0.001), and BO recovery point (*rho* = 0.32, *p* = 0.034).

### (ii) Smooth and step objective tests

**Fig 4:**
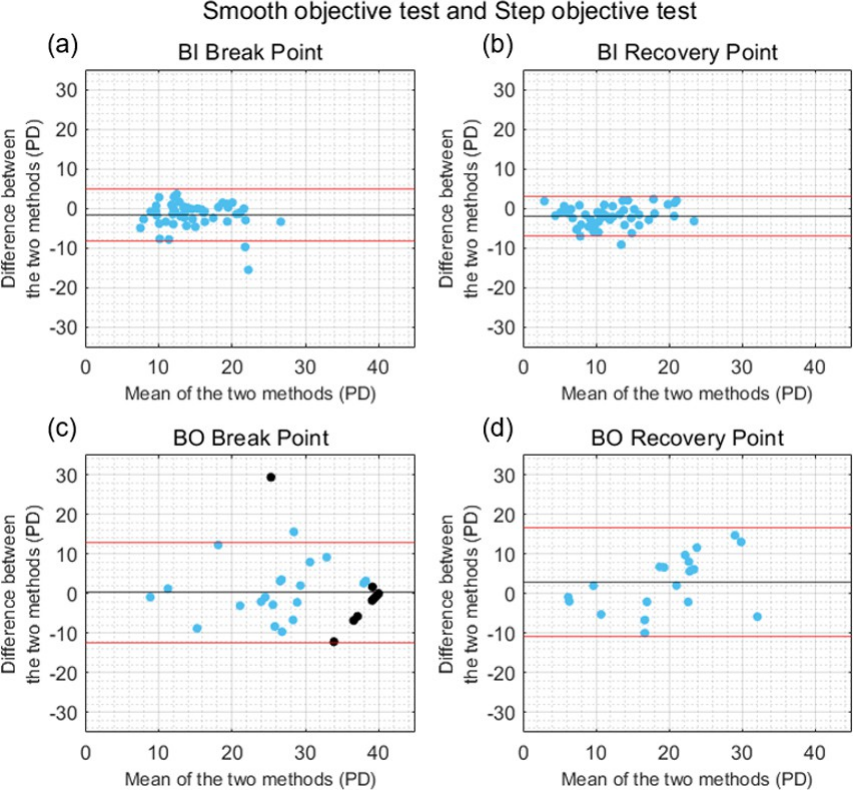
Bland-Altman plots comparing the results obtained with the smooth and step objective tests. (a) Base-In (BI) break point, (b) BI recovery point, (c) Base-Out (BO) break point, and (d) BO recovery point. Black horizontal lines correspond to the mean of the differences between tests, and red horizontal lines show the 95% limits of agreement. Black dots correspond to the participants who did not exhibit loss of motor fusion and were assigned a break point of 40 PD.

The mean ± SD of the differences in the BI break and recovery points measured with the smooth and step objective tests were -1.74 ± 3.35 PD and -1.97 ± 2.60 PD, respectively (Fig 4A and 4B). These differences were statistically significant (*U* = -3.47, *p <* 0.001, Cohen’s d = 0.51 for the BI break point; and *t*(48) = -5.31, *p* < 0.001, Cohen’s d = 0.75 for the BI recovery point). The agreement between the smooth and step tests for the BO break and recovery points was slightly better when the measure was objective than when it was subjective. The differences between the two objective tests were not statistically significant for the BO break point (0.31 ± 6.44 PD, *t*(48) = 0.34, *p* = 0.733, Cohen’s d = 0.04) nor for the BO recovery point (-2.84 ± 7.01 PD, *U* = -1.60, *p* = 0.108, Cohen’s d = 0.40) (Fig 4C and 4D). Only the 20 participants who exhibited loss of fusion during both objective tests were included in the BO recovery analysis. Despite the small mean difference between the two objective tests in the BO measurements, a high variability across subjects was found, as illustrated in Fig 4C and 4D. Strong significant correlations between the results of the smooth and step objective tests were found for the BI break point (*rho* = 0.81, *p* < 0.001), BI recovery point (*r* = 0.86, *p* < 0.001), BO break point (*r* = 0.75, *p* < 0.001) and BO recovery point (*rho* = 0.56, *p* = 0.001).

### (iii) Smooth subjective and objective tests

**Fig 5:**
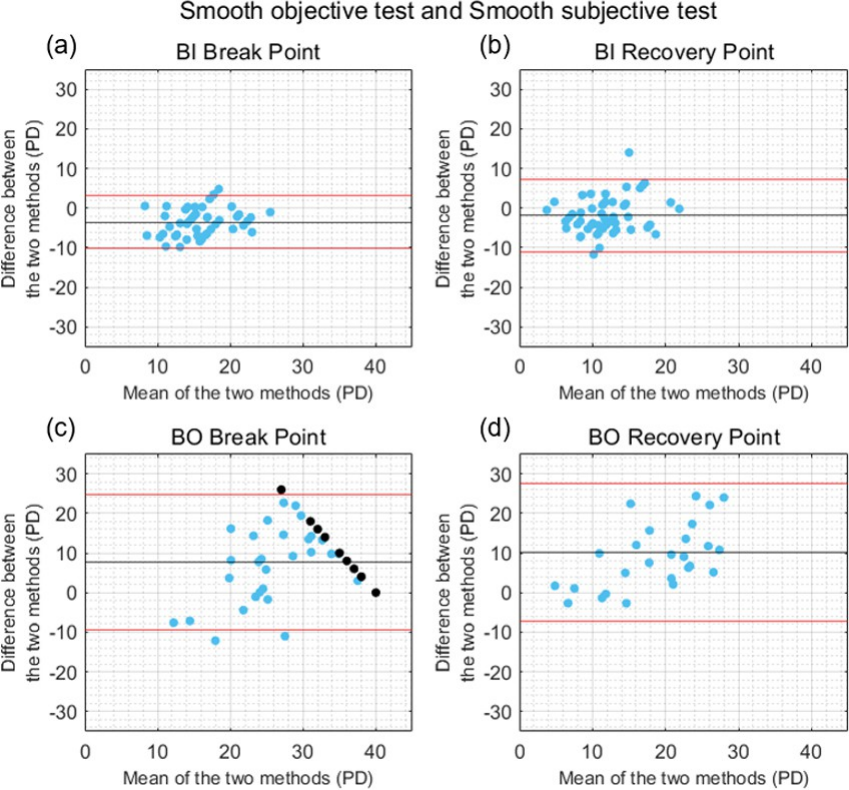
Bland-Altman plots comparing the results obtained with the smooth objective and subjective tests. (a) Base-In (BI) break point, (b) BI recovery point, (c) Base-Out (BO) break point, and (d) BO recovery point. Black horizontal lines correspond to the mean of the differences between tests, and red horizontal lines show the 95% limits of agreement. Black dots correspond to the participants who did not exhibit loss of motor fusion and were assigned a break point of 40 PD.

BI break points measured subjectively with the smooth test were significantly higher than those measured objectively with a mean difference ± SD of -3.58 ± 3.39 PD (*U* = -5.13, *p* < 0.001, Cohen’s d = 1.04) (Fig 5A). The mean ± SD of the differences in the BI recovery points measured with the two smooth tests of -1.86 ± 4.69 PD (Fig 5B) were at the limit of significance considering the adjusted significance level (*t*(48) = -2.78, *p* = 0.008, Cohen’s d = 0.39). A poorer agreement between the smooth objective and subjective tests was found for the BO break and recovery points. The mean ± SD of the differences in BO break and recovery points of 7.76 ± 8.74 PD and 10.12 ± 8.84 PD (Fig 5C and 5D) were statistically significant (*t*(48) = 6.21, *p* < 0.001, Cohen’s d = 0.88; and *U* = -4.43, *p* < 0.001, Cohen’s d = 1.14, respectively). The sample size for the BO recovery points comparison was reduced to the 28 participants who exhibited a BO break point with the two smooth tests. Moderate significant correlations between tests were found for the measures of BI break point (*rho* = 0.61, *p* < 0.001), BI recovery point (*r* = 0.49, *p* < 0.001), BO break point (*r* = 0.44, *p* = 0.001) and BO recovery point (*rho* = 0.46, *p* = 0.010).

### (iv) Step subjective and objective tests

**Fig 6:**
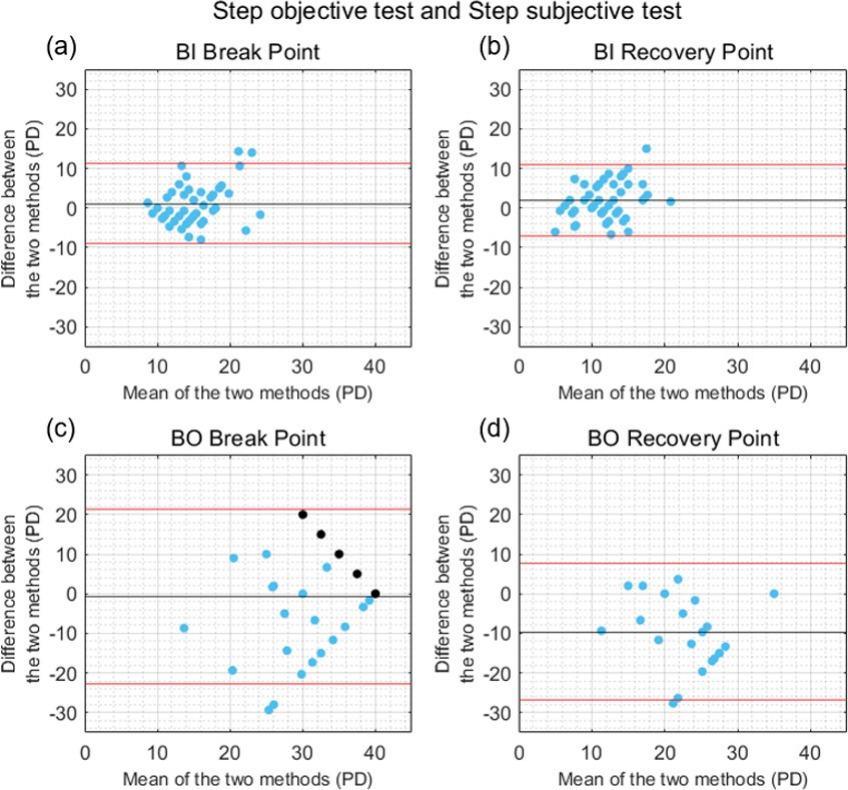
Bland-Altman plots comparing the results obtained with the step objective and subjective tests. (a) Base-In (BI) break point, (b) BI recovery point, (c) Base-Out (BO) break point, and (d) BO recovery point. Black horizontal lines correspond to the mean of the differences between tests, and red horizontal lines show the 95% limits of agreement. Black dots correspond to the participants who did not exhibit loss of motor fusion and were assigned a break point of 40 PD.

On average, the differences in the BI break point measured with the step objective and subjective tests (1.16 ± 5.10 PD) (Fig 6A) were not statistically significant (*U* = -1.25, *p* = 0.210, Cohen’s d = 0.22). For the BI recovery point, the differences between the two tests of 1.94 ± 4.66 PD (Fig 6B) reached statistical significance (*t*(48) = 2.92, *p* = 0.005, Cohen’s d = 0.41). Although, on average, the differences in the BO break point measured with the step objective and subjective tests were small and not significant (*U* = -0.19, *p* = 0.852, Cohen’s d = 0.06), a wide variability across participants was found (mean ± SD of the differences of -0.67 ± 11.21 PD) (Fig 6C). A similar variability across participants was found in the agreement between the two tests to measure the BO recovery point (mean ± SD of the differences of -9.59 ± 8.79 PD), and the differences were statistically significant (*U* = -3.58, *p* < 0.001, Cohen’s d = 1.09) (Fig 6D). In this case, 22 participants exhibited loss of fusion during the BO break point measurement and were included in the BO recovery comparison. The poorest correlations between tests were found for the pair of step tests, especially for the measures of BO break point (rho = 0.16, p = 0.259) and BO recovery point (rho = 0.13, p = 0.548). Low significant correlations between the step objective and subjective tests were found for the BI break point (rho = 0.34, p = 0.016) and BI recovery point (r = 0.38, p = 0.006).

## Discussion

In this experimental cross-sectional study, fusional vergence amplitudes were measured objectively by implementing two tests in an haploscopic set-up and measuring eye movements with a video-based eye-tracker. Fusional vergence amplitudes determined offline from the analysis of eye movements were compared with the results obtained with the conventional subjective smooth and step fusional vergence tests.

Using the same target size and method to evaluate fusional vergence amplitudes is recommended in order to obtain comparable results [15]. However, there is still noticeable variability of results even among studies with participants of comparable ages, and using similar target sizes, instrumentation, and procedures [15]. For example, Goss and Becker (2011) measured fusional vergence amplitudes in a group of pre-presbyopic adults with typical binocular vision using a target of the same size at the same viewing distance, same instrumentation, and similar experimental procedure as in the subjective tests in the present study [17]. On average, they obtained slightly larger negative fusional vergence amplitudes and considerably smaller positive fusional vergence amplitudes measured with the step fusional vergence test than in this study. The results of the smooth fusional vergence test were more similar in the two studies, especially for the positive fusional vergence amplitudes [17]. Antona et al. (2008) found smaller positive and negative fusional vergence amplitudes measured with the step fusional vergence test, and larger fusional vergence amplitudes measured with the smooth vergence test compared to the current study [16]. The differences between the results of these studies and the subjective tests in the current study could be partially explained by the fact that these authors did not seem to encourage participants to keep the stimulus single [16, 17]. In our study, they were instructed to do so, as it is typically done in clinics. This could have resulted in larger fusional vergence amplitudes than if participants would have been naïve and uninstructed to the task [29, 40].

In general, the results of the current study showed a better agreement between tests for the measure of negative fusional vergence amplitudes than for positive fusional vergence amplitudes (Figs 3–6). This is consistent with previous studies that compared the results obtained with the smooth and step fusional vergence tests using a Risley prism and a prism bar [16, 17, 29]. This may be related to the neural coupling between vergence and accommodation [30]. and the other stimuli which can affect convergence responses, and lead to increased variability between BO measures (e.g. proximal or tonic cues) [41, 42]. Importantly, similar levels of agreement were found between the smooth and step objective fusional vergence tests developed and implemented in this study. Although the differences in negative fusional vergence break and recovery points measured with the different tests were statistically significant for most of the four pairs of tests, the means of the differences were relatively close to 0, suggesting that there was not a clinically significant bias towards one method giving systematically larger or smaller results than the others. However, the large variability across participants led to wide 95% limits of agreement. Given this variability and the limited agreement found, it cannot be concluded whether step or smooth is the most appropriate procedure for vergence testing. While this is not the main objective of this study, it is an interesting question and further research is needed to better understand the impact of different vergence testing methodologies.

As indicated in the Results section, several participants did not report diplopia or exhibit loss of motor fusion during the measurement of positive fusional vergence amplitudes. This ceiling effect is clearly illustrated in Fig 2C by highly skewed distributions for the step subjective and smooth and step objective tests. The fact that these participants were assigned a break point of 40 PD could have led to an underestimation of the differences between tests. Twenty-seven participants did not exhibit loss of motor fusion during the step objective test, and only one of them did not report diplopia during the step subjective test. If these participants, shown in black in Fig 6C (many points are overlapped on top of each other), were excluded from the analysis and only those with a true measured break point were included, the mean difference ± SD in BO break point measured with the two tests was -7.87 ± 10.90 PD. For the other comparisons, the agreement results were qualitatively similar after excluding participants with an assigned break point.

In the standard assessment of fusional vergence amplitudes in clinics, before reporting diplopia, patients may also report blur vision, i.e., the blur point, which refers to the limit of the accommodative system to compensate for the increased vergence-driven accommodation to keep the stimulus focused [1, 6]. The blur point was not recorded in the current study as the haploscopic set-up did not include a system to measure accommodation objectively. Adding such instrumentation to the experimental set-up would allow us to measure blur points objectively and compare accommodation stability across tests. Appropriate control of accommodation, which was encouraged by using a small and high contrast stimulus and asking participants to keep the target clear, may have a role in reducing vergence response variability caused by accommodation instability, as previously reported for the measurement of heterophoria [43].

Despite the effort to unify the testing conditions across the four tests, such as testing distance or stimuli size, there were some methodological differences that could have contributed to the poor agreement between them. First, only one repetition was done to assess the fusional vergence amplitudes with the subjective tests. Doing three measurements would have unified the testing procedure between the objective and subjective tests and might have contributed to reduce the intra-subject variability in the subjective results [12, 14]. Secondly, in the subjective tests, the examiner started to decrease the prismatic power of the Risley prisms or the prism bar as soon as participants reported double vision, whereas in the objective tests, disparity always reached the maximum value of 40 PD. This methodological difference should have no effect on the measured fusional vergence break points, but could explain some differences between the recovery points measured with the objective and subjective tests. Perhaps the most relevant difference in measuring conditions between tests was the fact that, in the step subjective test, the prism bar placed in front of the right eye drove asymmetric vergence movements, whereas in the step objective test, the total disparity was divided into the two eyes resulting in symmetric vergence, i.e., along the sagittal plane. Further research is needed to quantify the impact of stimulating symmetric or asymmetric vergence on fusional vergence amplitudes. Thirdly, subjective tests were done in a light room and objective tests were done in a darkened room in order to improve eye-tracking signal reliability. Proximal cues present during the subjective tests performed in well illuminated conditions could have contributed to increased fusional vergence ranges, especially in base out conditions. However, the effect of proximal cues in closed-loop conditions is minimal, according to some studies [41, 44]. Finally, it should be noted that the initial study sample was of 49 participants, but on inspection of the vergence data, as aforementioned, some participants’ data were excluded from further analysis due to the lack of loss and recovery of fusion, which made certain comparisons unfeasible. With a sample size of 49 participants, an effect size of at least 0.40 is needed to achieve a statistical power of at least 0.8 [39]. Therefore, for most comparisons, the sample size of the study is acceptable as shown by the reported Cohen’s d in the Results section. However, given the high variability in the results of positive fusional vergence and the fact that several participants could not be included in the analyses of BO recovery points, the sample size is somewhat limited to draw final conclusions for some of the analyses.

In clinical practice, the fusional vergence test helps clinicians to diagnose binocular vision dysfunctions. The conventional testing procedures require non-expensive equipment and are relatively short. However, the results are subjective as they depend on the examiners and patients’ criteria and responses. This represents an important limitation, especially when testing populations for whom verbal responses cannot be obtained or are not reliable. In the current study, the feasibility of measuring fusional vergence amplitudes objectively has been demonstrated. The assessment of fusional vergence amplitudes from eye movements recordings overcomes the limitation of subjectivity and would provide clinicians with an additional tool to assess binocular function without the patients’ reports of diplopia and single vision, and with higher accuracy and resolution. Moreover, eye tracking recordings can provide new insights into the characteristics of eye movements in clinically relevant situations and the possibility to do more subtle analyses [45, 46]. The use of computerized set-ups allows to unify the testing conditions and control the visual stimulus characteristics to a greater extent, which could potentially lead to smaller inter-examiner variability and better repeatability [16]. Despite these advantages, the results of this study suggest that the poor agreement between the smooth and step fusional vergence tests cannot be explained exclusively by the subjectivity and non-uniform testing conditions, as similar levels of agreement were found for the objective tests. This suggests that intrinsic variability in the ocular motor system may also contribute to the poor agreement and repeatability of the fusional vergence tests. Further research is needed to implement objective tests using eye tracking systems in optometric clinical practice with simplified equipment, set-ups, and procedures suited to the general population which control adequately for the sources of variability, especially during fusional convergence assessment.

## Supporting information

S1 Data (XLSX)
